# Oxygen-Sensitivity and Pulmonary Selectivity of Vasodilators as Potential Drugs for Pulmonary Hypertension

**DOI:** 10.3390/antiox10020155

**Published:** 2021-01-21

**Authors:** Daniel Morales-Cano, Bianca Barreira, Beatriz De Olaiz Navarro, María Callejo, Gema Mondejar-Parreño, Sergio Esquivel-Ruiz, Jose A. Lorente, Laura Moreno, Joan A. Barberá, Ángel Cogolludo, Francisco Perez-Vizcaino

**Affiliations:** 1 Department of Pharmacology and Toxicology, School of Medicine, University Complutense of Madrid, 28040 Madrid, Spain; biancabarreira@med.ucm.es (B.B.); maria.callejo@ucm.es (M.C.); gemondej@ucm.es (G.M.-P.); sesquive@ucm.es (S.E.-R.); lmorenog@med.ucm.es (L.M.); acogolludo@med.ucm.es (Á.C.); fperez@med.ucm.es (F.P.-V.); 2 Instituto de Investigación Sanitaria Gregorio Marañón (IiSGM), 28007 Madrid, Spain; 3 Ciber Enfermedades Respiratorias (CIBERES), 28029 Madrid, Spain; joseangel.lorente@salud.madrid.org (J.A.L.); JBARBERA@clinic.cat (J.A.B.); 4 Centro Nacional de Investigaciones Cardiovasculares (CNIC), 28029 Madrid, Spain; 5 Hospital Universitario de Getafe, 28905 Madrid, Spain; bdolaiz@gmail.com; 6 Department of Pulmonary Medicine, Hospital Clínic-Institut d’Investigacions Biomèdiques August Pi i Sunyer (IDIBAPS), Universitat de Barcelona, 08036 Barcelona, Spain

**Keywords:** pulmonary hypertension, vascular, vasodilator, oxygen-sensing

## Abstract

Current approved therapies for pulmonary hypertension (PH) aim to restore the balance between endothelial mediators in the pulmonary circulation. These drugs may exert vasodilator effects on poorly oxygenated vessels. This may lead to the derivation of blood perfusion towards low ventilated alveoli, i.e., producing ventilation-perfusion mismatch, with detrimental effects on gas exchange. The aim of this study is to analyze the oxygen-sensitivity in vitro of 25 drugs currently used or potentially useful for PH. Additionally, the study analyses the effectiveness of these vasodilators in the pulmonary vs. the systemic vessels. Vasodilator responses were recorded in pulmonary arteries (PA) and mesenteric arteries (MA) from rats and in human PA in a wire myograph under different oxygen concentrations. None of the studied drugs showed oxygen selectivity, being equally or more effective as vasodilators under conditions of low oxygen as compared to high oxygen levels. The drugs studied showed low pulmonary selectivity, being equally or more effective as vasodilators in systemic than in PA. A similar behavior was observed for the members within each drug family. In conclusion, none of the drugs showed optimal vasodilator profile, which may limit their therapeutic efficacy in PH.

## 1. Introduction

Pulmonary hypertension (PH) is a disorder that affects the lung vasculature characterized by increased mean pulmonary arterial pressure [1]. Pulmonary arterial hypertension (PAH or group 1 PH) is a severe and progressive subtype of PH which involves primarily alterations in the small pulmonary arteries. A complex interplay among different factors in PAH are thought to contribute to the sustained vasoconstriction, cell proliferation, in situ thrombosis, inflammation and arterial wall stiffening, which result in reduced arterial lumen and increased pulmonary arterial pressure, leading to right ventricular hypertrophy and premature death [1,2,3]. Current targeted PAH therapy has increased survival, but mortality at 3 years after the diagnosis is still ≈ 30% [4].

PAH is characterized by an imbalance in tissue and circulating levels of vasoactive mediators. This imbalance includes a decrease in the production of vasodilators/growth inhibitors such as NO and prostacyclin, while there is an increase in the production of vasoconstrictor/pro-mitogens such as endothelin-1 (ET-1), thromboxane A_2_ (TXA_2_) and serotonin (5-HT) [5,6,7]. Current PAH treatments, based on drugs mimicking or inhibiting these endogenous vasoactive factors, are categorized into three classes: (1) prostacyclin and IP receptor agonists, (2) phosphodiesterase type 5 (PDE5) inhibitors and soluble guanylate cyclase (sGC) stimulators and (3) ET-1 receptor antagonists [8,9]. These therapies are limited by several factors. First, currently used vasodilators may exert their effects on poorly ventilated lung regions, leading to blood diversion to hypoxic alveoli with detrimental effects on gas exchange, i.e., ventilation-perfusion mismatch with increased shunt. This may be especially important in patients with underlying respiratory disease. Second, the constriction of pulmonary arteries (PA) is often resistant to vasodilators compared to systemic arteries. Thus, the use of high doses is accompanied by systemic vasodilation leading to side effects, including systemic hypotension. Third, the available drugs have focused mainly on vasodilation. While pulmonary vasodilation is a desired endpoint, the more severe forms of PAH are accompanied by vascular remodelling caused by inflammation, smooth muscle proliferation and vascular fibrosis.

An ideal drug to treat PH should show the following properties: (1) combine both vasodilator and antiproliferative effects, (2) exert selective vasodilator effects in the pulmonary circulation, avoiding systemic vasodilation, (3) exert selective vasodilator effects in the oxygenated lung areas, preserving hypoxic pulmonary vasoconstriction (HPV), (4) exert effective vasodilation under both Ca^2+^-dependent and Ca^2+^-independent pulmonary vasoconstriction and finally, (5) an appropriate pharmacokinetic (orally active and with prolonged half-life) and safety profile. In this regard, the expected benefits derived from its properties are the following: (1) reduced pulmonary arterial pressure, (2) in the long term reduced right ventricular hypertrophy and failure, (3) improved arterial oxygen saturation, decreasing the need for oxygen therapy, (4) improved clinical symptoms and quality of life, (5) increased exercise capacity and (6) increased survival.

Thus, in this in vitro study, we have characterized the drugs currently approved for PAH (except for ET-1 antagonists as discussed below), other agents that could be useful as potential therapies or drugs targeting major vasodilator pathways in order to compare: (1) their efficacy to relax pulmonary vessels compared to systemic arteries (pulmonary selectivity), and (2) whether their vasodilator effects are modified by the oxygen levels (oxygen selectivity).

## 2. Materials and Methods

The investigation conforms to the Royal Decree 1201/2005 and 53/2013 on the Care and Use of Laboratory Animals and all procedures were approved by our institutional Ethical Committee (ref. PROEX-251/15). Male Wistar rats (weight, 275 to 325 g; age, 12 weeks) were purchased from Harlan Iberica (Barcelona, Spain). Animals were kept under standard conditions of temperature 22 ± 1 °C and 12:12 h dark/light cycle and on standard rat chow and water ad libitum. Animals were sacrificed by decapitation under ketamine/xylazine (80 mg/8 mg/kg, ip) anesthesia.

The Human Research Ethics Committee of the Hospital Universitario de Getafe (Madrid, Spain) approved the use, after informed consent, of lung tissue discarded by pathologist following thoracic surgery (Ref. A04/16). Lung tissue was obtained from 26 patients (20 men, 6 women, mean age 67.0 ± 2.1 years) undergoing lobectomy or pneumonectomy during resection of lung carcinoma.

Reagents: Drugs and reagents were obtained from Sigma-Aldrich Quimica (Alcobendas, Spain), except retigabine (Axon, Groningen, The Netherlands), GW0742 (Tocris, Bristol, UK), riociguat (Chemexpress, Shangai, China), treprostinil and imatinib mesilate (Cayman, MI, USA) and rosiglitazone (Selleck Chemicals, Munich, Germany). Drugs were dissolved in water or in DMSO and final vehicle concentrations were ≤ 0.1%.

Vascular reactivity: Intrapulmonary artery rings (2–3 mm long, 0.5–0.8 mm internal diameter) were dissected from human or rat lung tissue, and mesenteric arteries (MA) rings (2–3 mm long, 0.5–0.8 mm internal diameter) were dissected from the rat mesenteric bed. To analyze the vasodilator effects of the different drugs in vitro, preparations were mounted in a wire myograph in Krebs buffer solution maintained at 37 °C. The chamber was bubbled with 95% O_2_ and 5% CO_2_ (oxygen) or 95 % N_2_ and 5% CO_2_ (hypoxia), yielding a 2–4% O_2_ in the buffer due to contact with room air [10]. After equilibration, arterial rings were firstly stimulated with KCl (80 mM) and then washed. After another equilibration period of 20 min, arterial rings were contracted with a cocktail of endothelin-1 (3·10^−9^ M), the TXA_2_ mimetic U46619 (3·10^−8^ M) and 5-HT (3·10^−5^ M). At these concentrations, the agents were submaximally effective, inducing ≈ 50% of their individual maximal response and together they induced a strong sustained vasoconstrictor response (99.9 ± 5.9% of the response to KCl). When a plateau contraction was reached, the different vasodilating drugs were added in a cumulative fashion.

Statistical analysis: Results are expressed as means ± standard deviation (SD) or standard error of the mean (SEM) of n measurements where n identifies the number of animals or patients. Individual cumulative concentration response curves were fitted to a logistic equation. The drug concentration that induced 50% relaxation (IC_50_) was calculated from this equation. For the concentration response of vasodilators, the maximal responses (E_max_) were expressed as percent of inhibition of the contraction induced by the mixture of vasoconstrictors. The pIC_50_ was defined as the negative logIC_50_ (concentration of the drug that produces 50% inhibition of the contraction). We defined pulmonary selectivity as the ratio of IC_50_ values in MA and the IC_50_ values in PA both under oxygen conditions. Oxygen selectivity was defined as the ratio of IC_50_ value for PA under hypoxia and the IC_50_ value for PA under oxygen. Whenever a drug did not reach a 50% relaxation for all experimental conditions, we used the IC_30_ and pIC_30_. Statistical analyses were performed comparing drug-induced responses using an unpaired Students’ *t*-test. A value of *p* < 0.05 was considered statistically significant.

## 3. Results

### 3.1. Efficacy and Potency as Vasodilators

**Figure 1:**
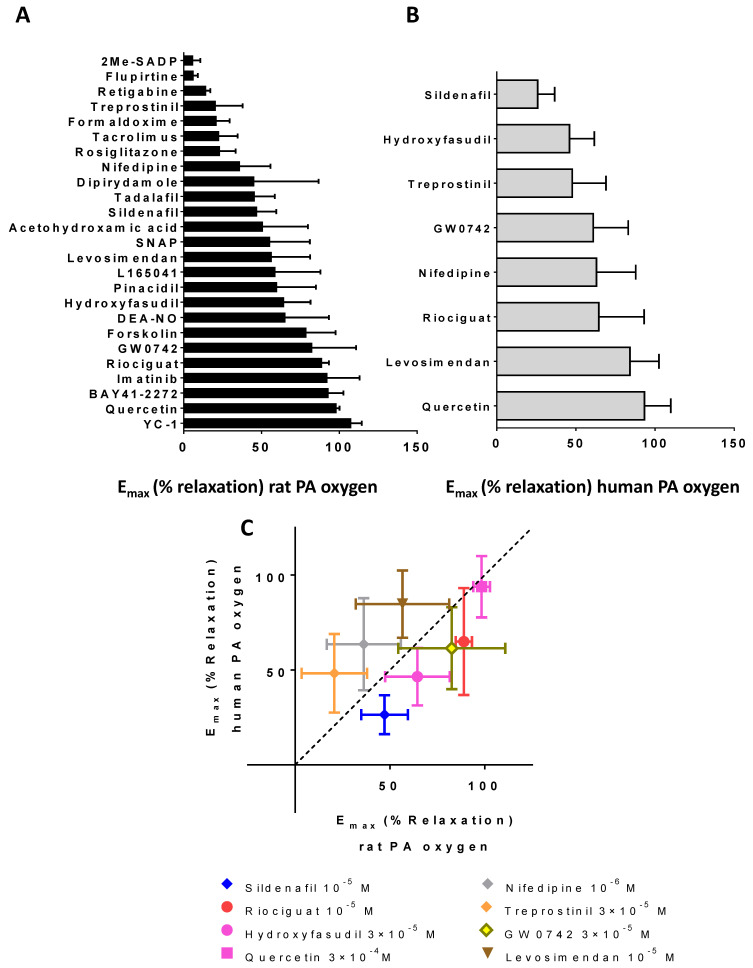
Efficacy of vasodilators. Bar graph representing the percent of maximal relaxation (E_max_) of several vasodilator drugs on contractile tone in rat PA arteries (**A**) and human PA (**B**) bubbled with 95% O_2_-5% CO_2_ (PA oxygen). Comparison of the E_max_ effects of several drugs in rat and human PA (**C**). Abscissa, vasodilation of rat PA (E_max_ (% relaxation)); ordinate, vasodilation on human PA (E_max_ (% relaxation)). Calculated from data in Figures S1–S8. Data are means ± SD.

The vasodilator effects of the drugs in PA under hypoxia or high oxygen conditions or in MA, as a prototype of a systemic vessel, are shown in Figures S1–S8 and the Emax values for the relaxation of rat and human PA in high oxygen conditions by these drugs summarized in Figure 1A,B. The parameters regarding efficacy (i.e., E_max_) and potency (pIC_50_) derived from these concentration-response curves in rat and human arteries are listed in Table 1 and Table 2. The pIC_30_ values were also calculated for some drugs which did not achieve the 50% relaxation for all conditions (Table 3).

The flavonoid quercetin, the sGC stimulators (riociguat, BAY41-2272 and YC-1) and imatinib were the most efficacious to relax rat PA, inducing almost full relaxation. An intermediate efficacy (50–85% relaxation) was observed for levosimendan, the PPARβδ agonists GW0742 and L165041, the adenylyl cyclase activator forskolin, the NO donors SNAP, DEA-NO and acetohydroxamic acid, the ROCK1/2 inhibitor hydroxyfasudil and the KATP activator pinacidil. A third group producing 40–50% relaxation included the PDE5 inhibitors sildenafil, tadalafil and dipyridamole and the Ca^2+^ channel blocker nifedipine. Finally, the weaker vasodilators (<30%) included the PPARγ agonist rosiglitazone, the prostacyclin analogue teprostinil, the Kv7 agonists flupirtine and retigabine, the calcineurin inhibitor tacrolimus or the P2Y1 agonist 2-Me-SADP.

A subset of the vasodilators was further analyzed in human PA (Figure 1B). Intermediate-high efficacy (60–90% relaxation) was observed also for quercetin, riociguat, levosimendan, nifedipine and GW0742. Teprostinil and hydroxyfasudil produced approx. 50% maximal relaxation. Finally, sildenafil produced a weak relaxant response. Thus, in Figure 1C, the correlation analysis yielded a significant direct association (*p* < 0.05) between the efficacy in rats and humans (r^2^ of 0.47 and a slope of 0.61). However, there are some differences. The dotted line drawn from 0 to 100% separates the drugs with preferential effects in humans (i.e., drugs appearing above the dashed line) from those with preferential effect in rats (i.e., drugs below the dashed line). It shows that the efficacy of teprostinil, levosimendan and nifedipine was higher in human PA while sildenafil, riociguat, hydroxyfasudil and GW0742 were better vasodilators in rat PAs.

### 3.2. Pulmonary and Oxygen Selectivity

**Figure 2:**
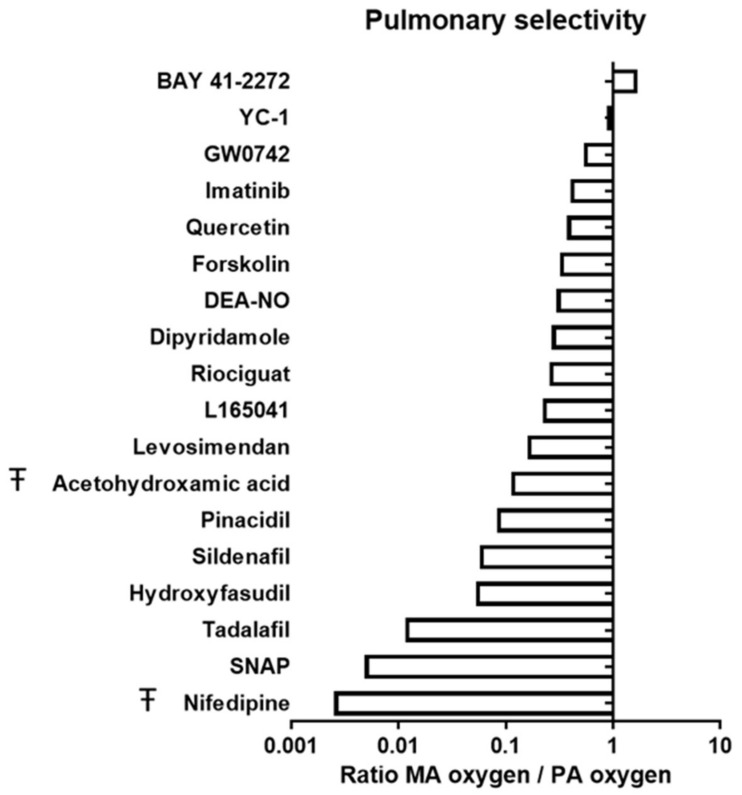
Selectivity of drugs for pulmonary vessels. Relative pulmonary selectivity of different vasodilator drugs in rat arteries. Data are expressed as relative potency (pIC_50_) in MA vs. PA under high oxygenation. Ŧ indicates that data are expressed as relative pIC_30_ values in MA vs. PA. Values > 1 indicate greater selectivity for PA oxygen; while values < 1 indicate greater selectivity for MA oxygen.

Figure 2 plots the pulmonary selectivity index, i.e., the ratio of the IC_50_ or IC_30_ values in MA and PA. It shows that none of the studied drugs was pulmonary selective, i.e., the index was not significantly higher than one for any drug. Nifedipine, SNAP and tadalafil exerted marked systemic selectivity (pulmonary selectivity index ≤ 0.01). Mild systemic selectivity (0.02–0.1) was found for hydroxyfasudil, sildenafil, pinacidil and acetohydroxamic acid. L165041, riociguat, DEA-NO, forskolin, quercetin and imatinib were moderately (0.2–0.7) but significantly more potent in MA. Finally, BAY41-2272, YC-1 and GW0742 were equipotent in MA and PA.

**Figure 3:**
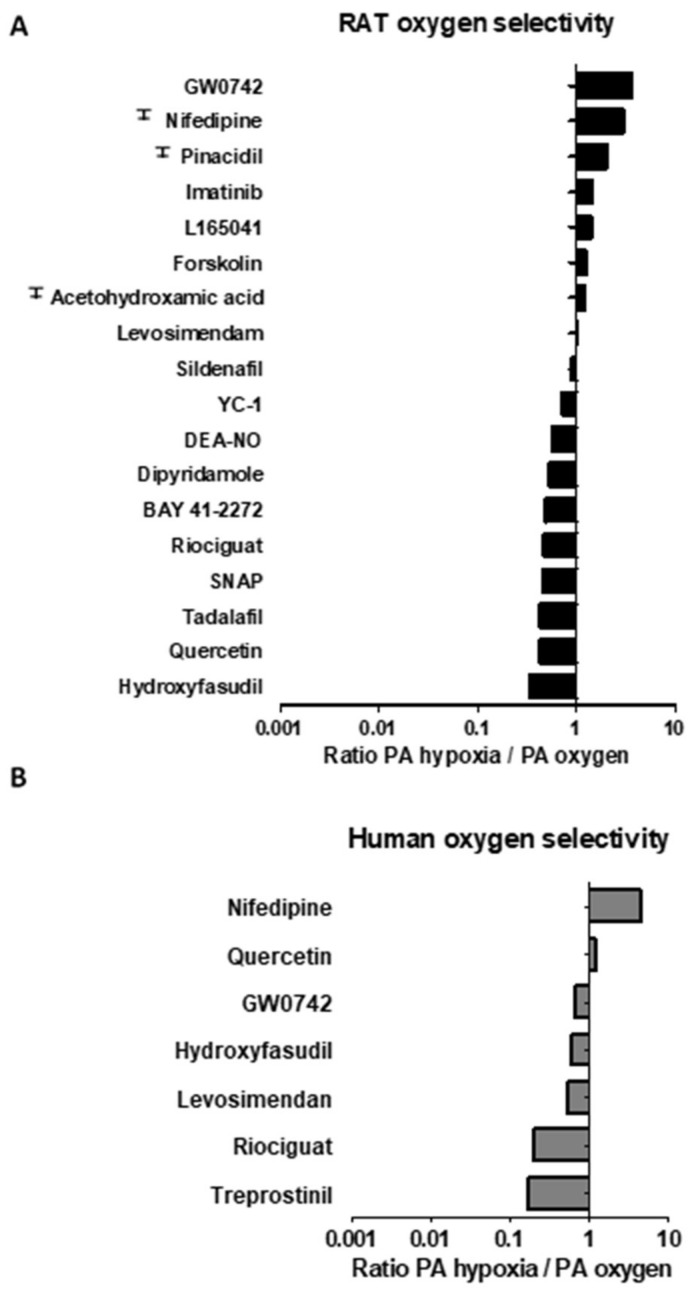
Relative oxygen selectivity of different vasodilator drugs in rat (**A**) and human (**B**) PA. Data are expressed as relative potency (pIC_50_) in PA under conditions of high vs. low oxygenation, respectively. Ŧ indicates that data are expressed as relative pIC_30_ values in PA under high vs. low oxygenation. Values > 1 indicate selectivity for PA oxygen; while values < 1 indicate selectivity for PA hypoxia.

Regarding oxygen selectivity in rat and human PA (Figure 3A,B), most drugs were similarly effective under high oxygen or hypoxia. The only drug which showed oxygen selectivity was GW0742, i.e., was significantly more potent (3.7 fold) as vasodilator under high oxygen than under low levels of oxygen in rat PA. Unfortunately, this oxygen selectivity of GW0742 was not observed in human PA (Figure 3B). On the other hand, hydroxyfasudil, quercetin, riociguat and BAY41-2272 were more potent under hypoxia than under high oxygen conditions in rat PA. This preferential effect under hypoxic conditions was also observed in human PA for riociguat but not for hydroxyfasudil or quercetin. Despite an apparent trend in Figure 3A,B for nifedipine being pulmonary selective in rat and human PA, its effects were not statistically different when compared under oxygen vs. hypoxic conditions.

## 4. Discussion

In this study, we have characterized a wide range of existing vasodilators in order to compare their efficacy to relax pulmonary vessels, their selectivity for the pulmonary circulation and their oxygen-sensitivity. This is by far the largest study of this kind.

Increased vascular resistance in PAH results from functional narrowing due to PA vasoconstriction and from anatomical narrowing of the PA due to endothelial and smooth muscle cell proliferation. In the clinic, the contribution of vasoconstriction to the overall increase in pulmonary vascular resistance is generally assessed by the acute vasodilator response to NO, adenosine or a prostacyclin analogue or the efficacy of chronic vasodilator treatment [11]. Collectively, the contribution of the vasoconstrictor component is thought to be important in the early phases while vascular cell proliferation seem to be more important in the late stages of the disease [12,13]. However, it has been reported that rats treated with SU5416 plus hypoxia for three weeks and kept for 32 weeks in normoxia still maintain high pressure despite pulmonary vascular remodeling being significantly reduced, suggesting that pulmonary vasoconstriction may be also important in the long term [14].

As noted above, current PAH therapies are focused mainly on vasodilation either by enhancing vasodilator mechanisms such as the prostacyclin-cAMP pathway and the NO-cGMP pathway or by inhibiting vasoconstrictor mechanisms such as endothelin-1 and Ca^2+^ entry pathways [8,9]. Thus, under the conditions used herein, we found marked differences in efficacy to induce pulmonary vasodilation among the different drugs. We have put to the test different vasodilators in rat and human PA under conditions of a strong vasoconstrictor stimulus and we found that only the flavonoid quercetin, the sGC stimulators and imatinib (a tyr-kinase inhibitor) caused a full relaxant response. Levosimendan, GW0742 and L165041 (the PPARβδ agonists), forskolin (an adenylyl cyclase activator), SNAP, DEA-NO and acetohydroxamic acid (NO donors), hydroxyfasudil (a ROCK1/2 inhibitor), pinacidil (a KATP activator), the PDE5 inhibitors and nifedipine (a Ca^2+^ channel blocker) caused an intermediate relaxation. By contrast, rosiglitazone (a PPARγ agonist), treprostinil (a prostacyclin analogue), flupirtine and retigabine (Kv7 agonists), tacrolimus (a calcineurin inhibitor) and 2-Me-SADP (a P2Y1 agonist) caused a weak vasodilation. Overall, the fact that vasoconstriction may be resistant to NO-donors or nifedipine but sensitive to other vasodilators challenges the concept that PAH with a lack of response to acute inhaled NO or to chronic nifedipine, i.e., the so called non vasoreactive PH, does not involve pulmonary artery vasoconstriction [11]. The fact that some vasodilators fail to lower pulmonary arterial pressure does not necessarily mean that vasoconstriction does not play a role. A lack of a vasodilator response may only reflect the choice of an inadequate drug [15,16].

Taken together, the low efficacy of vasodilators modulating Ca^2+^ channel activity, either directly (Ca^2+^ channel blockade with nifedipine) or indirectly (via K^+^ channel opening induced hyperpolarization with pinacidil, retigabine or flupirtine), suggests that the vasoconstriction of PA, at least under our experimental conditions, is not mainly mediated by Ca^2+^ entry through CaL channels. Ca^2+^-independent pathways mediated by ROCK or other protein kinases, including protein kinase C, mitogen-activated protein kinase or non-receptor tyrosine kinases seem to play a large role in PA contraction [17,18]. Thus, we observed that the most clinically used drugs: PDE5 inhibitors, prostacyclin analogues and nifedipine, produced only partial relaxant effects, whereas sGC stimulators produced strong relaxant responses which nearly abolished the vasoconstrictor cocktail-induced vasoconstriction. These data are in good agreement with the effects observed previously by us and other authors on the NO-cGMP pathway. Thus, while PDE5 inhibitors show weak vasodilator effects in human PA [19] or newborn piglet PA [20], the sGC stimulators induces a strong concentration-dependent relaxation in ovine PA [21], and piglet PA [22]. Therefore, these results suggest that, in general, stimulation of sGC may have a theoretical advantage over PDE5 inhibition because of its NO-independent mechanism of action [23]. Moreover, the vasodilator effects of drugs modulating ion channels were not significantly different under high vs. low oxygen concentrations. This is consistent with the poor effects of calcium antagonist on gas exchange [24].

As mentioned above, the ideal vasodilator drug for the treatment of PH should present selectivity for pulmonary over systemic vessels. However, we found that none of the studied drugs was pulmonary selective. In this study, both PDE5 inhibitors and sGC stimulators display no pulmonary selectivity in vitro, showing similar or stronger effects in MA than in PA. PDE5 inhibitors have been proposed to act mainly as pulmonary vasodilators since they reduce pulmonary pressure in patients with PAH, and lack of significant systemic vasodilation [25], but our results suggest that they are able to induce a similar vasodilation in PA and MA. In addition, consistent with prior reports, the poor pulmonary selectivity of the three sGC stimulators showed in our study was comparable with results observed in several animal models [26,27] and in patients with PAH, distal chronic thromboembolic PH or PH associated with mild to moderate interstitial lung disease [28] and in patients with PH associated with COPD [29] where this family of compounds did not demonstrated pulmonary selectivity. Finally, the poor pulmonary selectivity of the calcium antagonist nifedipine is in good agreement with results previously presented by our group in piglet MA and PA, where nifedipine induces a stronger relaxant responses in MA than in PA [30] and in patients with PH secondary to advanced COPD and patients with PAH, where administration of nifedipine induces preferential systemic vasodilation with no significant changes in pulmonary arterial pressures [31]. In addition, Kv7 channel and KATP channel activators, which indirectly reduce calcium entry via membrane hyperpolarization, showed poor efficacy in pulmonary vessels. However, it should be noted that these drugs may show enhanced efficacy to relax PA from animal models of PH [32,33].

The main aim of the present study was to identify oxygen-sensitive vasodilators (i.e., vasodilators which act less effectively under hypoxic conditions). The identification of this kind of drug would be of great importance. They would benefit not only PAH patients with hypoxemia but especially patients in group 3 PH, i.e., PH associated with respiratory diseases (e.g., chronic obstructive pulmonary disease and interstitial lung disease). In patients with these conditions the use of vasodilators is debated because of their potential detrimental effects on gas exchange, produced by the inhibition of HPV [34,35]. Oxygen sensitive vasodilators would be expected to preserve HPV and improve oxygenation. In addition, oxygen-sensitive drugs would be useful for patients with acute lung injury which show severe hypoxemia and increased pulmonary arterial pressure. We found that only the PPARβδ agonist GW0742 was more effective in PA in high oxygen conditions than in hypoxia. The difference was mild (3.7-fold) but significant. Unfortunately, this was not reproduced in human PA. Similarly, GW0742 was not more effective in mouse PA contracted by hypoxia compared to PA contracted by U44619 or phenylephrine [36]. Moreover, another PPARβδ agonist L165041 was not oxygen sensitive in PA. All these data limit the possible relevance of oxygen selectivity of GW0742.

Because the results of the in vitro studies may be strongly influenced by the experimental conditions, we must consider several methodological issues and limitations of the present study. First, we have not analyzed the effects of the widely used endothelin receptor antagonists because due to the peculiarities of the in vitro assays, the effects of these drugs would be strongly determined by the concentration of endothelin added to the media. Second, we have studied the effects of several vasodilators under conditions of high pulmonary arterial tone, which presumably reflects what happens in PH. A mixture of the vasoconstrictors ET-1, 5-HT and TXA_2_ mimetic U46619 was chosen as the contractile stimulus since all of these factors have been associated with PH [5,6,7]. Third, arteries from each systemic vascular bed may respond differently to drugs. We chose MA of a similar diameter than PA as representative systemic arteries. Fourth, drugs were initially screened in control rat arteries and only selected drugs were further analyzed in human arteries. Fifth, the vasodilator analysis was performed in arteries from healthy rats and from patients that did not have any clinical evidence of PH. However, we have studied the effects of vasodilators under conditions of high vascular tone induced by relevant vasoconstrictors, which presumably reflects what happens in PH. Sixth, the study is mainly descriptive, a mechanistic study was planned for oxygen-sensitive drugs but unfortunately none of the studied ones were oxygen selective in human PA.

## 5. Conclusions

In conclusion, there were large differences in the efficacy and potency of relaxation of both rat and human PA demonstrated by different classes of vasodilators we tested. Unfortunately, none of the drugs showed oxygen-sensitivity or pulmonary selectivity. These results highlight the need for better pulmonary vasodilator drugs, which could be useful in other groups of PH different from PAH, especially in PH associated to respiratory diseases and hypoxia.

## Figures and Tables

**Table 1:** Parameters derived from the concentration-response curves in rat pulmonary and mesenteric arteries.

Drug	Rat Mesenteric Artery			Rat Pulmonary Artery					
				Hypoxia			Oxygen		
	pIC_50_	E_max_ *	*n*	pIC_50_	E_max_	*n*	pIC_50_	E_max_ *	*N*
Sildenafil	6.18 ± 0.59	≥74.6 ± 22.7 ^a^	5	4.98 ± 1.08	≥49.9 ± 20.7	5	4.94 ± 1.44	≥47.1 ± 12.3	7
Tadalafil	7.01 ± 0.59 ^a^	≥84.9 ± 17.6 ^a^	4	5.44 ± 0.92	≥51.8 ± 21.0	3	5.08 ± 0.80	≥45.7 ± 12.8	4
Dipyridamole	5.37 ± 0.27	≥62.6 ± 19.1	3	5.07 ± 0.37	≥51.5 ± 13.5	3	4.80 ± 0.71	≥45.4 ± 41.3	3
YC-1	6.38 ± 0.19	110.5 ± 11.3	5	6.46 ± 0.17	107.4 ± 4.9	6	6.32 ± 0.15	107.7 ± 16.5	6
BAY 41-2272	7.42 ± 0.26	91.9 ± 17.1	6	7.96 ± 0.17 ^c^	111.4 ± 10.0	6	7.65 ± 0.20	93.1 ± 23.4	6
Riociguat	7.45 ± 0.19 ^b^	107.8 ± 12.8 ^a^	6	7.19 ± 0.21 ^c^	89.9 ± 14.5	6	6.86 ± 0.09	89.0 ± 4.3	6
Hydroxyfasudil	6.03 ± 0.13 ^b^	95.1 ± 3.7 ^b^	7	5.23 ± 0.27 ^d^	≥70.2 ± 26.0	7	4.75 ± 0.19	≥64.5 ± 17.0	8
Imatinib	5.70 ± 0.22 ^b^	101.9 ± 20.5	5	5.13 ± 0.18	≥88.3 ± 18.2	5	5.30 ± 0.17	≥92.3 ± 20.8	6
Quercetin	5.71 ± 0.09 ^b^	102.3 ± 6.7	6	5.65 ± 0.16 ^d^	111.9 ± 12.7 ^c^	6	5.28 ± 0.10	98.3 ± 4.4	6
GW0742	5.44 ± 0.20	94.3 ± 20.3	7	4.60 ± 0.22 ^d^	≥57.9 ± 32.8	9	5.17 ± 0.29	≥82.6 ± 28.3	10
L165041	5.27 ± 0.26 ^b^	94.1 ± 34.0	8	4.47 ± 0.29	≥50.0 ± 21.1	7	4.62 ± 0.22	≥59.0 ± 28.9	7
Rosiglitazone	4.78 ± 0.19	≥72.6 ± 2	4	N/A	≥39.2 ± 2.9	4	N/A	≥23.3 ± 10.1	4
SNAP	6.57 ± 0.71 ^b^	81.9 ± 29.3	4	4.60 ± 0.29	≥61.9 ± 13.0	4	4.25 ± 0.58	≥55.6 ± 25.5	4
DEA-NO	6.24 ± 0.17 ^b^	94.7 ± 18.7	5	5.96 ± 0.16	83.9 ± 19.6	5	5.71 ± 0.16	≥65.4 ± 28.0	3
Acetohydroxamic acid	3.70 ± 0.28	≥68.2 ± 12.9	3	N/A	≥44.8 ± 10.8	3	N/A	≥51.0 ± 28.9	3
Formaldoxime	N/A	≥43.1 ± 20.1	5	N/A	≥47.1 ± 22.9	5	N/A	≥21.2 ± 8.4	3
Treprostinil	6.05 ± 0.41	≥61.9 ± 28.2 ^b^	7	N/A	≥24.5 ± 23.0	6	N/A	≥20.6 ± 17.3	8
Forskolin	7.25 ± 0.07 ^b^	96.6 ± 5.5	3	6.65 ± 0.05	≥83.2 ± 3.1	3	6.76 ± 0.08	≥78.9 ± 18.7	3
Nifedipine	8.78 ± 0.27	97.3 ± 13.8 ^b^	8	N/A	≥28.3 ± 20.2	8	N/A	≥36.2 ± 19.6	9
Retigabine	5.14 ± 0.18	93.0 ± 10.0 ^b^	3	N/A	≥8.1 ± 6.4	3	N/A	≥14.4 ± 2.7	3
Flupirtine	4.92 ± 0.48	≥70.1 ± 18.7 ^b^	3	N/A	≥5.6 ± 12.3	3	N/A	≥6.4 ± 2.7	3
Pinacidil	6.49 ± 0.24 ^b^	102.3 ± 3.0 ^b^	5	N/A	≥46.4 ± 23.5	5	5.40 ± 0.15	≥60.0 ± 25.0	5
2Me-SADP	N/A	≥15.5 ± 11.8	4	N/A	≥25.4 ± 21.2	4	N/A	≥6.1 ± 4.6	4
Tacrolimus	5.01 ± 1.04	≥50.39 ± 16.7 ^a^	3	N/A	≥37.2 ± 5.4	3	N/A	≥22.8 ± 11.9	3
Levosimendan	6.28 ± 0.28	91.3 ± 16.2 ^a^	4	5.47 ± 0.62	≥67.1 ± 24.4	7	5.48 ± 0.18	≥56.6 ± 24.6	15

Values are expressed as mean ± SD, *n* indicates the number of experiments from different animals assessed. Data were obtained from the individual cumulative concentration-response curves fitted to a logistic equation as shown in supplementary figures and compared using unpaired Student’s *t*-test. N/A, indicates that the 50% effect was not achieved precluding the calculation of the pIC_50_. ^a^ and ^b^ indicate *p* < 0.05 and *p* < 0.01, respectively, mesenteric vs. pulmonary artery oxygen. ^c^ and ^d^ indicate *p* < 0.05 and *p* < 0.01, respectively, pulmonary artery hypoxia vs. pulmonary artery oxygen. * The ≥ symbol indicates that E_max_ was not achieved at the maximal drug concentration tested; the data indicate the relaxation achieved at the maximal drug concentration and the true E_max_ may be equal or higher to the one shown in the table.

**Table 2:** Parameters derived from the concentration-response curves in human pulmonary arteries under hypoxia or oxygen conditions.

Drug	Human Pulmonary Artery Hipoxia			Human Pulmonary Artery Oxygen		
	pIC_50_	E_max_ *	*n*	pIC_50_	E_max_ *	*n*
Hydroxyfasudil	4.80 ± 0.59	≥55.2 ± 28.0	6	4.58 ± 0.53	≥46.5 ± 15.2	5
Nifedipine	5.16 ± 0.57	≥53.9 ± 21.9	6	5.82 ± 0.62	≥63.5 ± 24.3	6
Sildenafil	5.09 ± 1.05	≥48.0 ± 9.9 ^a^	4	N/A	≥26.4 ± 10.3	5
Quercetin	4.47 ± 0.27	88.8 ± 33.5	6	4.56 ± 0.14	93.8 ± 16.2	8
GW0742	4.79 ± 0.38	≥69.0 ± 31.4	3	4.63 ± 0.15	≥61.5 ± 21.6	5
Levosimendan	6.54 ± 0.49	74.8 ± 35.3	5	6.27 ± 0.24	84.7 ± 17.7	5
Riociguat	6.62 ± 0.23 ^b^	≥79.3 ± 17.4	11	5.93 ± 0.39	≥65.9 ± 28.2	10
Treprostinil	5.55 ± 0.37	≥58.0 ± 26.9	3	4.79 ± 0.52	≥48.2 ± 20.8	5

Values are expressed as mean ± SD, *n* indicates the number of experiments from different animals assessed. N/A indicates that the 50% effect was not achieved precluding the calculation of the IC_50_. Data were obtained from the individual cumulative concentration-response curves fitted to a logistic equation and compared using unpaired Student’s *t*-test. ^a^ and ^b^ indicate *p* < 0.05 and *p* < 0.01, respectively, pulmonary artery hypoxia vs. pulmonary artery oxygen. * The ≥ symbol indicates that E_max_ was not achieved at the maximal drug concentration tested; the data indicate the relaxation achieved at the maximal drug concentration and the true E_max_ may be equal or higher to the one shown in the table.

**Table 3:** pIC_30_ values calculated from the concentration-response curves induced by different drugs in rat arteries.

Drug	Rat Mesenteric Artery		Rat Pulmonary Artery			
			Hypoxia		Oxygen	
	pIC_30_	*n*	pIC_30_	*N*	pIC_30_	*n*
Rosiglitazone	5.18 ± 0.20 ^b^	4	4.65 ± 0.10	4	N/A	4
Acetohydroxamic acid	4.60 ± 0.29 ^a^	3	3.56 ± 0.28	3	3.65 ± 0.36	3
Formaldoxime	4.85 ± 0.68	5	4.63 ± 0.36	5	N/A	3
Treprostinil	6.97 ± 0.47	7	5.39 ± 0.81	6	N/A	8
Nifedipine	9.03 ± 0.37 ^b^	8	5.96 ± 1.16	8	6.43 ± 0.54	9
Pinacidil	6.90 ± 0.30 ^b^	5	5.78 ± 0.39	5	6.10 ± 0.33	5
Tacrolimus	6.78 ± 0.68	3	5.61 ± 0.33	3	N/A	3

Values are expressed as mean ± SD, *n* indicates the number of experiments from different lung samples assessed. N/A indicates that the 50% effect not achieved precluding the calculation of the IC_50_. Data were obtained from the individual cumulative concentration-response curves fitted to a logistic equation and compared using unpaired Student’s *t*-test. ^a^ and ^b^ indicate *p* < 0.05 and *p* < 0.01, respectively, mesenteric artery vs. pulmonary artery oxygen.

## Data Availability

Data is contained within the article or Supplementary Materials.

## Supplementary Materials

The following are available online at https://www.mdpi.com/2076-3921/10/2/155/s1; Figure S1: Effects of NO donors on vascular tone. Figure S2: Effects of PDE5 inhibitors on vascular tone. Figure S3: Effects of sGC stimulators on vascular tone. Figure S4: Effects of drugs targeting ion channels on vascular tone. Figure S5: Effects of adenylate cyclase activators on vascular tone. Figure S6: Effects of PPAR agonists on vascular tone. Figure S7: Effects of kinase inhibitors on vascular tone. Figure S8: Effects of drugs used for other indications on vascular tone.
