# Supplementary material for: Oxygen-Sensitivity and Pulmonary Selectivity of Vasodilators as Potential Drugs for Pulmonary Hypertension
Source: Antioxidants (Basel). 2021 Jan 21;10(2):155. doi: 10.3390/antiox10020155 (PMC7911835; doi:10.3390/antiox10020155)
Supplement: Supplementary file 1 [file antioxidants-10-00155-s001.pdf]

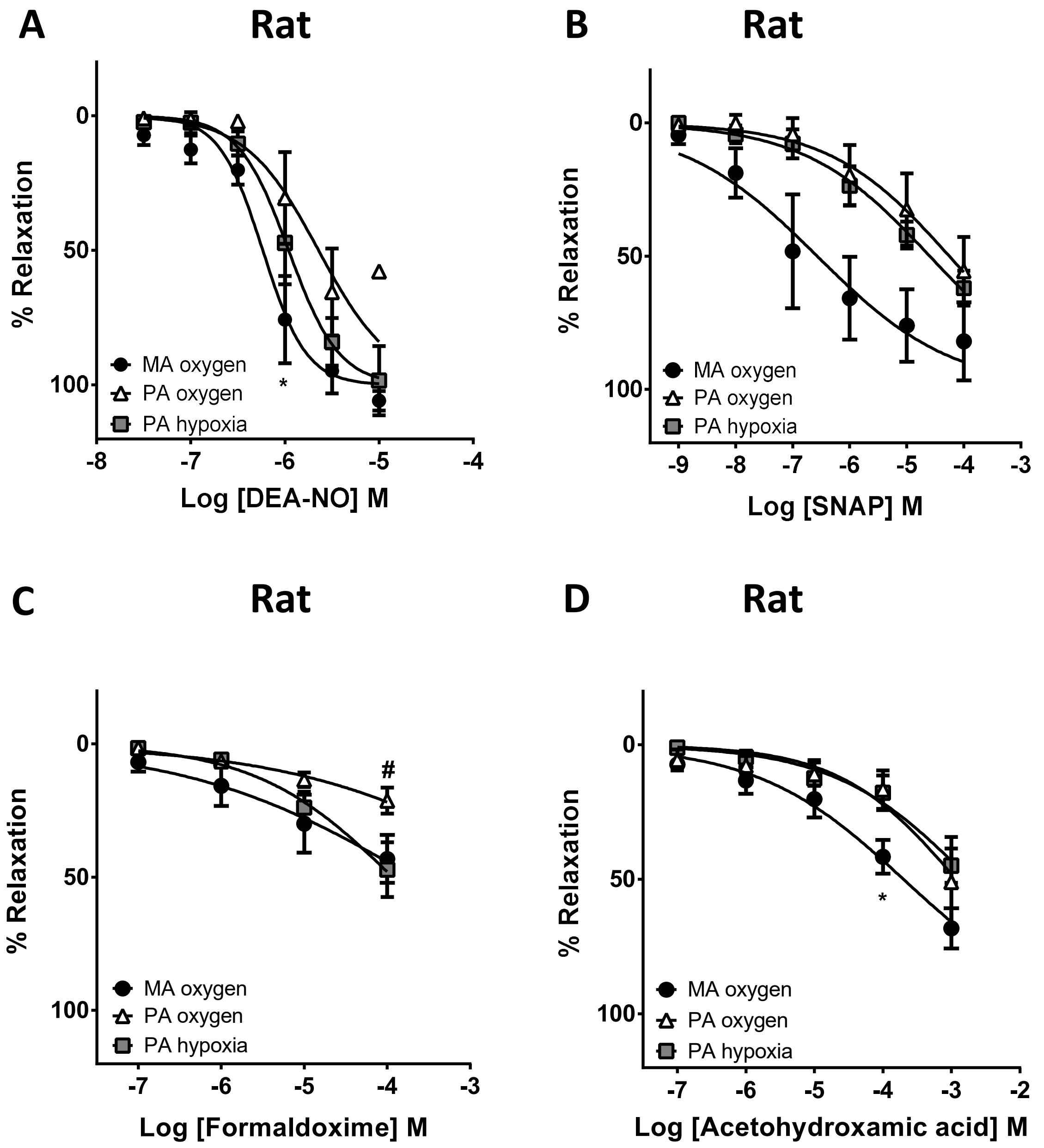

Supplementary figure 1: Effects of NO donors on vascular tone. Concentration dependent relaxation induced by the NO donors SNAP (A; n=4), DEA-NO (B; n=3-5), formaldoxime (C; n=3-5) and acetohydroxamic acid (D; n=3) in rat mesenteric arteries (MA oxygen) and PA under high oxygen (PA oxygen) or hypoxic (PA hypoxia) conditions. Arteries were initially stimulated with a cocktail of U46619 ($3x10^{-8}$ M), 5-HT ($3x10^{-6}$ M) and ET-1 ($3x10^{-9}$ M). Results are means ± SEM. Drug induced responses were compared with PA oxygen using two-way ANOVA followed by Bonferroni post hoc test. * indicates P<0.05 MA oxygen vs PA oxygen. # indicates P<0.05 PA hypoxia vs PA oxygen.

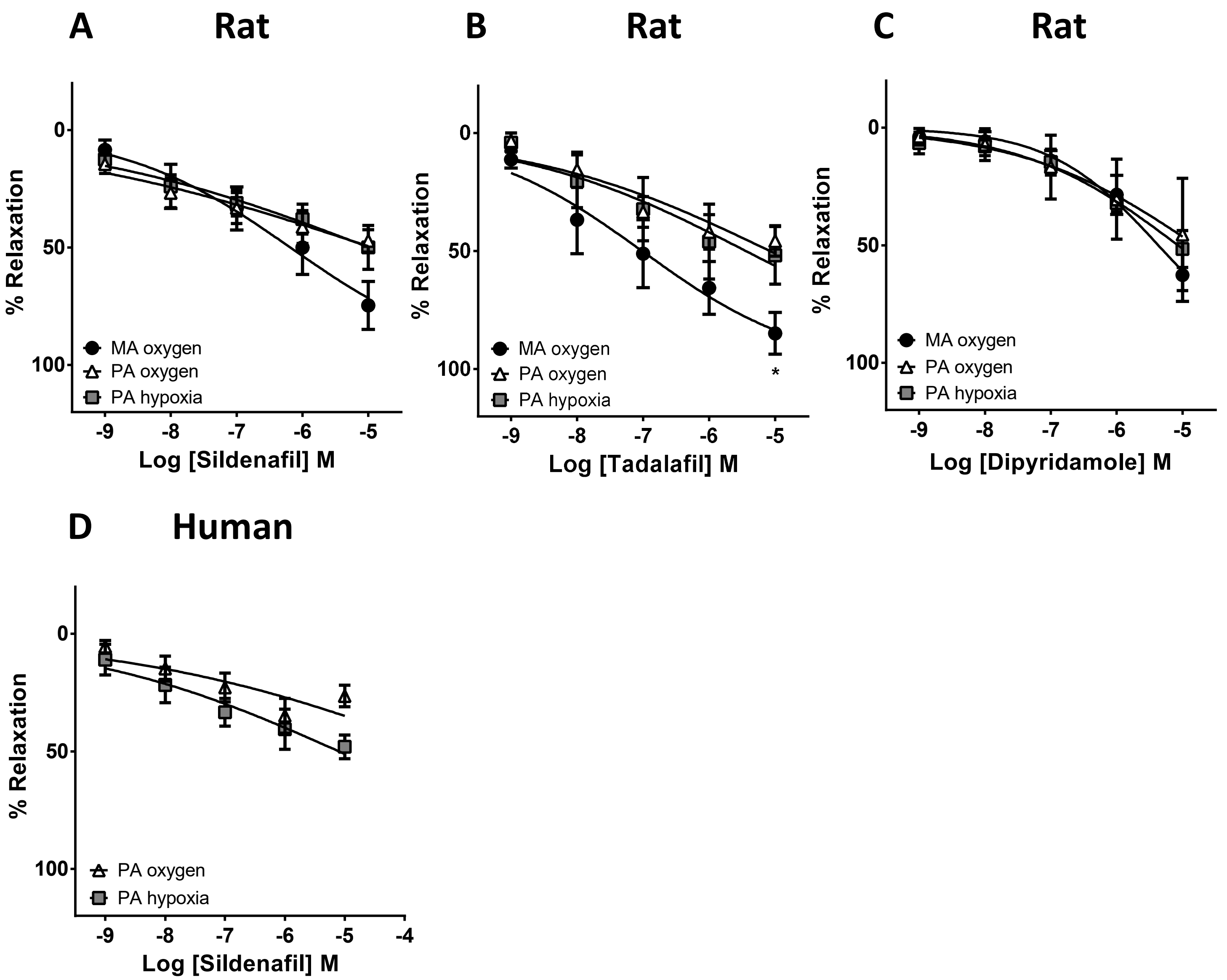

Supplementary figure 2: Effects of PDE5 inhibitors on vascular tone. Concentration-dependent relaxation induced by the PDE5 inhibitors sildenafil (A; n=5-7) and tadalafil (B; n=3-4), and the nonselective PDE inhibitor dipyridamole (C; n=3) in rat mesenteric arteries (MA oxygen) and PA under high oxygen (PA oxygen) or hypoxic (PA hypoxia) conditions. Concentration-dependent relaxation induced by the PDE5 inhibitors sildenafil (D; n=4-5) in human PA high oxygen (PA oxygen) or hypoxic (PA hypoxia) conditions. Arteries were initially stimulated with a cocktail of U46619 ($3x10^{-8}$ M), 5-HT ($3x10^{-6}$ M) and ET-1 ($3x10^{-9}$ M). Results are means ± SEM. Drugs induced responses were compared with PA oxygen using two-way ANOVA followed by Bonferroni post hoc test for A, B and C and unpaired Student's t-test for D. * indicates $P<0.05$ MA oxygen vs PA oxygen.

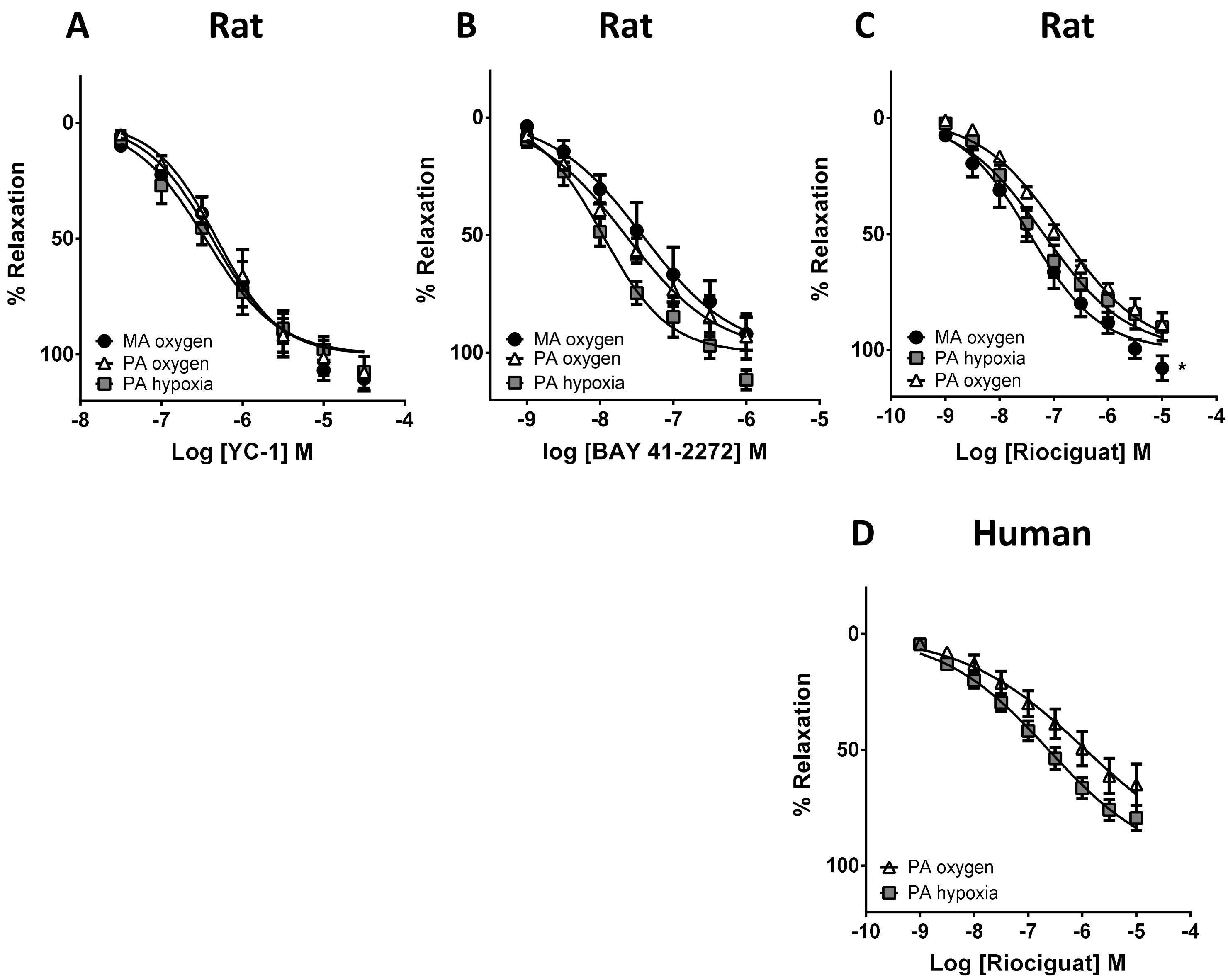

Supplementary figure 3: Effects of sGC stimulators on vascular tone. Concentration-dependent relaxation induced by the sGC stimulators YC-1 (A; n=5-6), BAY 41-2272 (B; n=6) and riociguat (C; n=6) in rat mesenteric arteries (MA oxygen), PA under high oxygen (PA oxygen) or hypoxic (PA hypoxia) conditions. Concentration-dependent relaxation induced by the sGC stimulator riociguat (D; n=10-11) in human PA under high oxygen (PA oxygen) or hypoxic (PA hypoxia) conditions. Arteries were initially stimulated with a cocktail of U46619 ($3x10^{-8}$ M), 5-HT ($3x10^{-6}$ M) and ET-1 ($3x10^{-9}$ M). Results are means ± SEM. Vasodilator responses were compared with PA oxygen using two-way ANOVA followed Bonferroni post hoc test for A, B and C and unpaired Student's t-test for D. * indicates P<0.05 MA oxygen vs PA oxygen

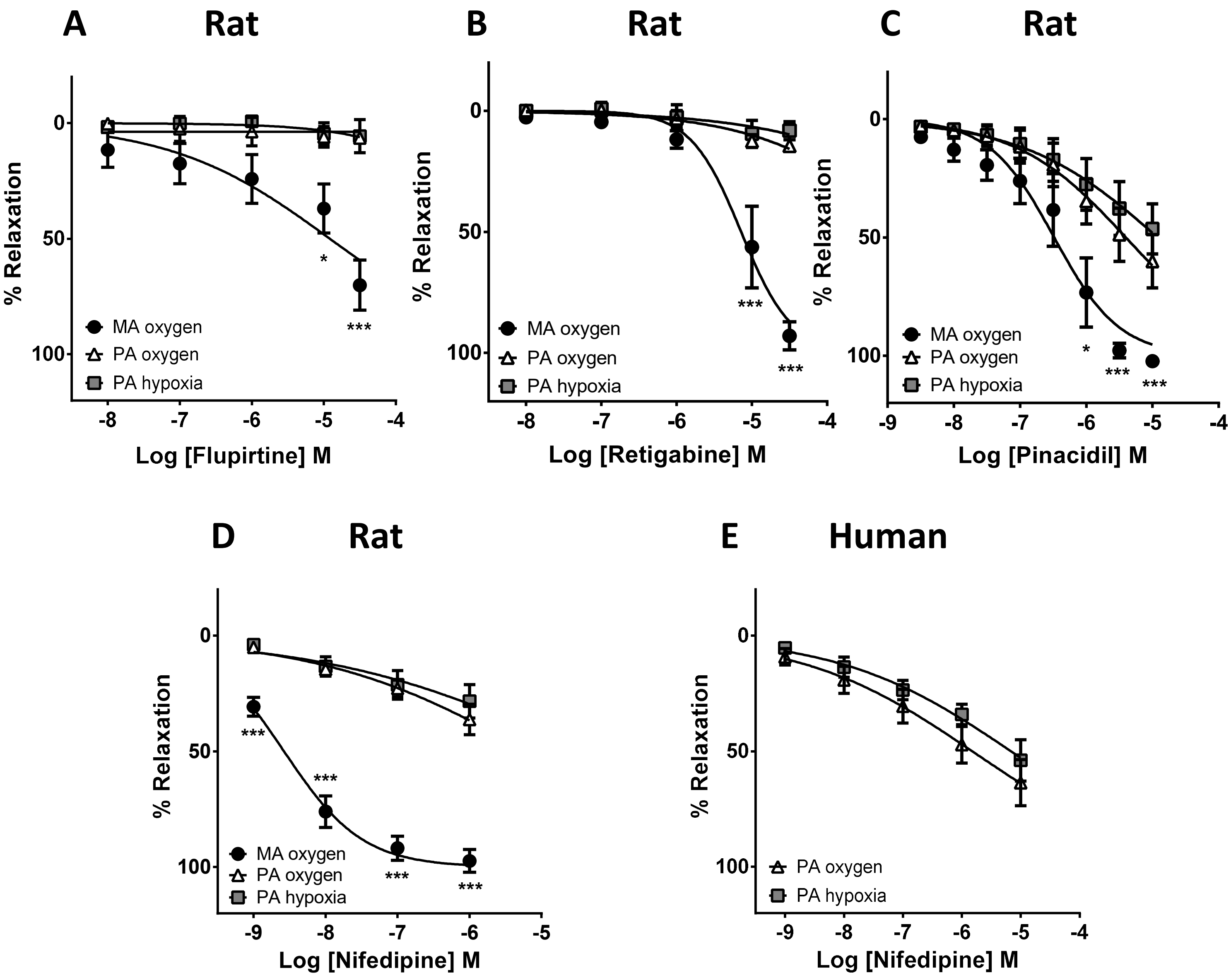

Supplementary figure 4: Effects of drugs targeting ion channels on vascular tone. Concentration-dependent relaxation induced by the Kv7 channels agonists flupirtine (A; n=3) and retigabine (B; n=3), the ATP-sensitive potassium channel activator pinacidil (C; n=5) and the CCB nifedipine (D; n=8-9) in rat MA (MA oxygen) and PA under high oxygen (PA oxygen) or hypoxic (PA hypoxia) conditions. Concentration-dependent relaxation induced by nifedipine (E; n=6) in human PA under high oxygen (PA oxygen) or hypoxic (PA hypoxia) conditions. Arteries were initially stimulated with a cocktail of U46619 ($3x10^{-8}$ M), 5-HT ($3x10^{-6}$ M) and ET-1 ($3x10^{-9}$ M). Results are means ± SEM. Vasodilator responses were compared with PA oxygen using two-way ANOVA followed Bonferroni post hoc test for A, B, C, E and F and unpaired Student's t-test for D. * and *** indicates P<0.05 and P< 0.001 MA oxygen vs PA oxygen.

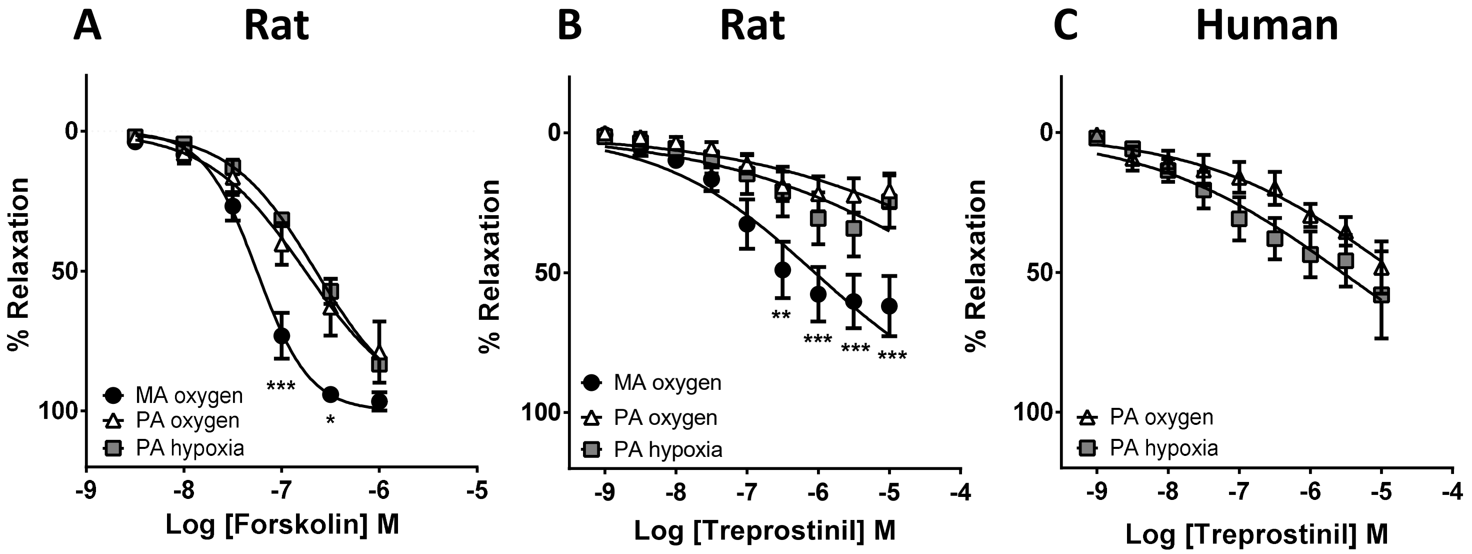

Supplementary figure 5: Effects of adenylate cyclase activators on vascular tone. Concentration-dependent relaxation induced by the PGI2 mimetic treprostinil (A; n=6-8) and the adenylate cyclase activator forskolin (B; n=3) in rat MA (MA oxygen) and PA under high oxygen (PA oxygen) or hypoxic (PA hypoxia) conditions. Concentration-dependent relaxation induced by treprostinil (C; n=6) in human PA under high oxygen (PA oxygen) or hypoxic (PA hypoxia) conditions. Arteries were initially stimulated with a cocktail of U46619 ($3x10^{-8}$ M), 5-HT ($3x10^{-6}$ M) and ET-1 ($3x10^{-9}$ M). Results are means ± SEM. Vasodilator responses were compared with PA oxygen using two-way ANOVA followed by Bonferroni post hoc test for A and B and unpaired Student's t-test for C. *, ** and ***indicate P<0.05, P<0.01 and P<0.001 MA oxygen vs PA oxygen.

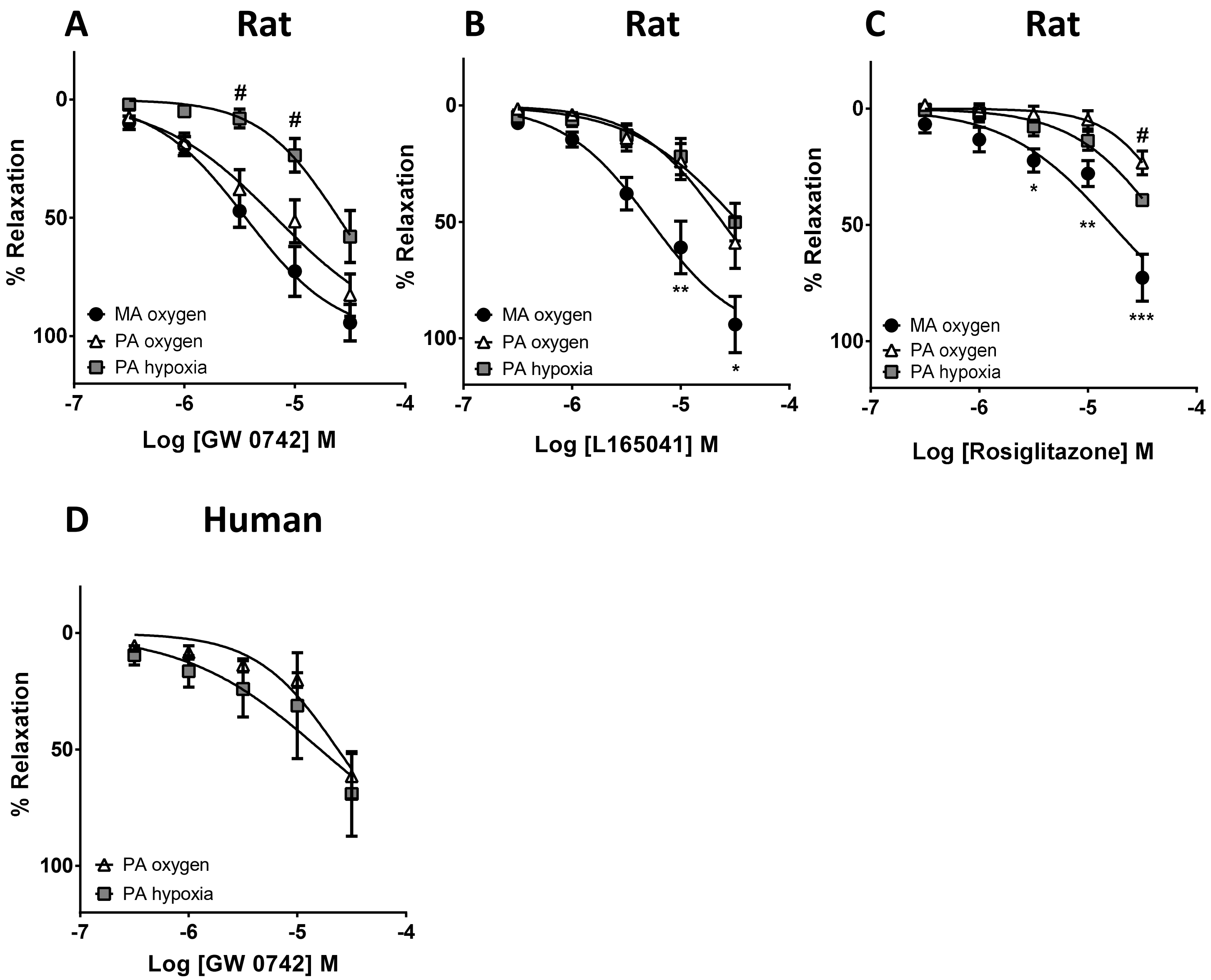

Supplementary figure 6: Effects of PPAR agonists on vascular tone. Concentration-dependent relaxation induced by the PPARβ/δ agonists GW0742 (A; n=7-10) and L165041 (B; n=7-8) and the PPARγ agonist rosiglitazone (C; n=4) in rat MA arteries (MA oxygen) and PA under high oxygen (PA oxygen) or under hypoxic (PA hypoxia) conditions. Concentration-dependent relaxation induced by the PPARβ/δ agonist GW0742 (C; n=3-5) in human PA under high oxygen (PA oxygen) or hypoxic (PA hypoxia) conditions. Arteries were initially stimulated with a cocktail of U46619 ($3x10^{-8}$ M), 5-HT ($3x10^{-6}$ M) and ET-1 ($3x10^{-9}$ M). Results are means ± SEM. Vasodilator induced responses were compared with PA oxygen using two-way ANOVA followed by Bonferroni post hoc test for A, B and C and unpaired Student's ttest for D. *, **and *** indicate P<0.05, P< 0.01 and P<0.001 MA oxygen vs PA oxygen. # indicates P<0.05 PA hypoxia vs PA oxygen.

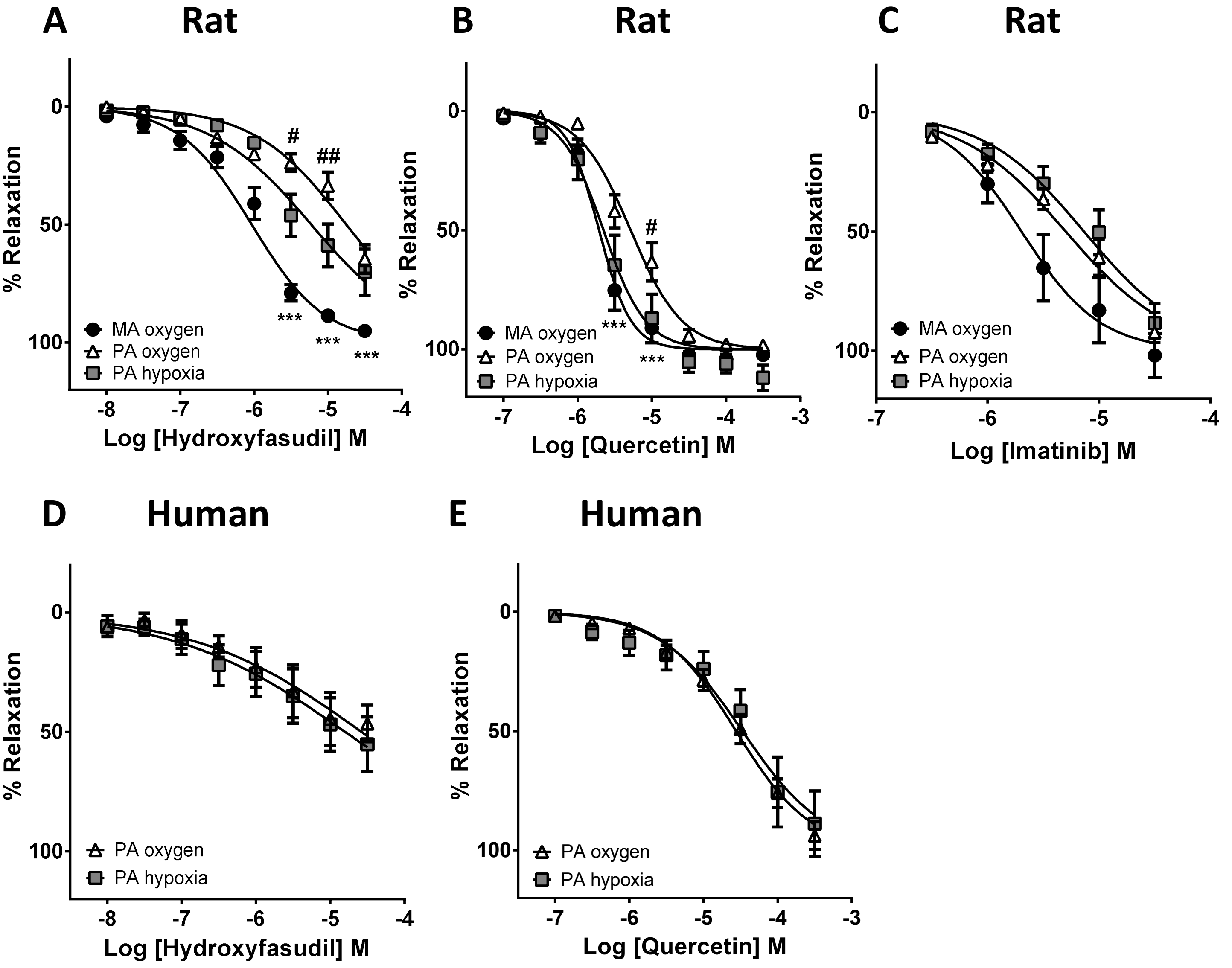

Supplementary figure 7: Effects of kinase inhibitors on vascular tone. Concentration-dependent relaxation induced by the ROCK inhibitor hydroxyfasudil (A; n=7-8), the flavonoid quercetin (B; n=6) and the tyr-kinase inhibitor imatinib (C; n=5-6) in rat MA (MA oxygen) and PA under high oxygen (PA oxygen) or hypoxic (PA hypoxia) conditions. Concentration-dependent relaxation induced by the ROCK inhibitor hydroxyfasudil (D; n=5-6) and the flavonoid quercetin (E; n=6-8) in human PA under high oxygen (PA oxygen) or hypoxic (PA hypoxia) conditions. Arteries were initially stimulated with a cocktail of U46619 ($3x10^{-8}$ M), 5-HT ($3x10^{-6}$ M) and ET-1 ($3x10^{-9}$ M). Results are means ± SEM. Vasodilator responses were compared with PA oxygen using two-way ANOVA followed by Bonferroni post hoc test for A, B and C and unpaired Student's t-test for D and E. *** indicates P<0.001 MA oxygen vs PA oxygen. # and ## indicate P<0.05 and P<0.01 PA hypoxia vs PA oxygen.

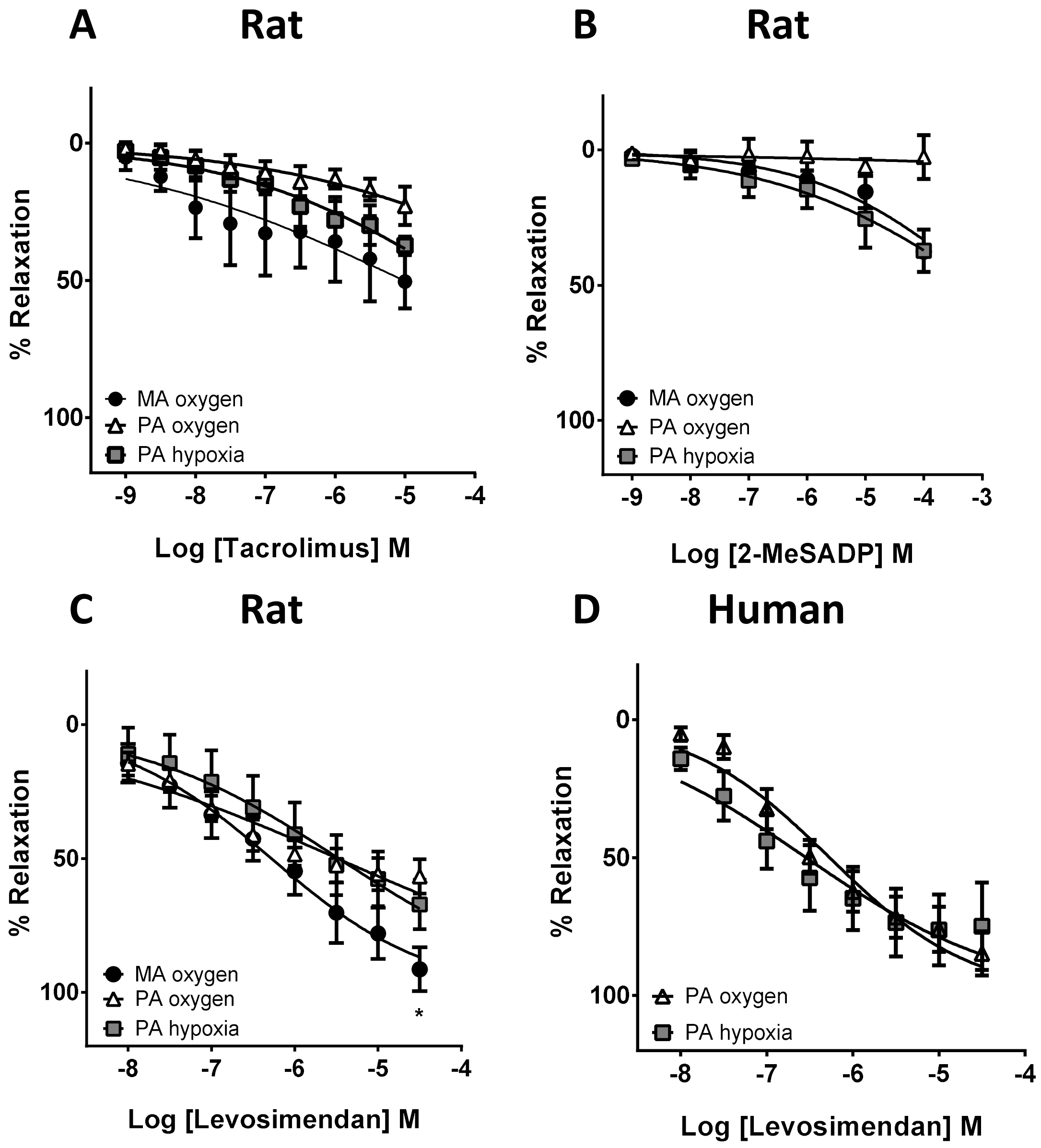

Supplementary figure 8: Effects of drugs used for other indications on vascular tone. Concentration dependent relaxation induced by the calcineurin inhibitor tacrolimus (A; n=3), the P2Y1 receptor agonist 2-MeSADP (B; n=4) and the calcium sensitizer levosimendan (C; n=4-15) in rat MA (MA oxygen) and PA under high oxygen (PA oxygen) or hypoxic (PA hypoxia) conditions. Concentration-dependent relaxation induced by the calcium sensitizer levosimendan (D; n=5) in human PA under high oxygen (PA oxygen) or hypoxic (PA hypoxia) conditions. Arteries were initially stimulated with a cocktail of U46619 ($3x10^{-8}$ M), 5-HT ($3x10^{-6}$ M) and ET-1 ($3x10^{-9}$ M). Results are means ± SEM. Vasodilators responses were compared with PA oxygen using two-way ANOVA followed by Bonferroni post hoc test for A, B and C and unpaired Student's t-test for D. * indicates P< 0.05 MA oxygen vs PA oxygen.
